# LncRNA LOXL1-AS is up-regulated in thoracic aortic aneurysm and regulated proliferation and apoptosis of aortic smooth muscle cells

**DOI:** 10.1042/BSR20191649

**Published:** 2019-09-13

**Authors:** Ben Huang, Shuyang Lu, Hao Lai, Jun Li, Yongxin Sun, Chunsheng Wang

**Affiliations:** Department of Cardiovascular Surgery, Zhongshan Hospital, Fudan University, Xietu Road No. 1609, Shanghai City 200032, P.R. China

**Keywords:** apoptosis, lncRNA Giver, lncRNA LOXL1-AS, proliferation, thoracic aortic aneurysm

## Abstract

Long non-coding RNA LOXL1-AS is up-regulated in several types of cancers. The present study was carried out to explore the potential interactions between LOXL1-AS and lncRNA Giver in thoracic aortic aneurysm (TAA). We found that LOXL1-AS was up-regulated in TAA patients than in healthy controls in aortic media specimens. Altered expression levels of LOXL1-AS distinguished TAA patients from healthy controls. LncRNA Giver was also up-regulated in TAA patients than in healthy controls in aortic media specimens, and was positively correlated with LOXL1-AS. LOXL1-AS overexpression mediated the up-regulation of Giver in human aortic smooth muscle cells, while Giver overexpression failed to significantly affect LOXL1-AS. LOXL1-AS and Giver overexpression resulted in promoted proliferation and inhibited apoptosis of HAOSMC. Giver silencing played an opposite role and attenuated the effect of LOXL1-AS overexpression. Therefore, LOXL1-AS was up-regulated in TAA and regulated proliferation and apoptosis of LOXL1-AS by up-regulating Giver.

## Introduction

Thoracic aortic aneurysm (TAA), which is the supra-diaphragmatic aorta dilatations caused by expansion and weakening of the arterial wall, has been recognized as a leading cause of deaths [1]. Genetic factors play critical roles in the development of TAA [2,3] (Mizuguchi et al. 2004; Schwarze et al. 2001). It has been reported that about 20% of nonsyndromic TAA patients have affected first-degree relatives [2,3]. Besides genetic factors, aging, hypertension, arteriosclerosis, and autoimmune diseases of inflammatory that affect the aorta also contribute to the occurrence of TAA [4]. Moreover, genetic factors can also interact with other risk factors to participate in TAA [5], indicating the complex pathogenesis of this disease.

Long non-coding RNAs (lncRNAs, longer than 200 nts) are a group of RNA transcripts lacking protein coding capacity [6]. Recent clinical and experimental studies have demonstrated lncRNAs as critical determinants in human diseases [7,8], including TAA [9,10]. Therefore, regulation of lncRNA expression may assist the prevention and treatment of TAA. However, function of only a few lncRNAs has been characterized in TAA [9,10], which limits their clinical applications. It has been reported that lncRNA Giver participates in inflammation, oxidative stress, and proliferation in vascular smooth muscle cells [11], which are involved in TAA [12], indicating its involvement in TAA. Our preliminary deep sequencing data suggested up-regulated expression of Giver in TAA, and its positive correlation with lncRNA LOXL1-AS. The present study was therefore carried out to explore the potential interactions between these two lncRNAs in TAA.

## Materials and methods

### Patients

Our study included 50 patients with TAA and 50 healthy controls in Zhongshan Hospital, Fudan University during the time period of January 2016 and May 2018. Patients’ inclusion criteria: (1) patients with no history of malignancies; (2) patients received no treatment before admission; and (3) newly diagnosed cases. Exclusion criteria: (1) patients transferred from other hospitals; (2) patients who received any treatment within 3 months before admission; and (3) patients complicated with other clinical disorders. The diameter of TAA ranged from 3.6 to 4.9 cm, with a mean of 4.4 ± 0.5 cm. The 50 patients included 28 males and 22 females, and age ranged from 38 to 63 years and the mean age was 46.8 ± 5.2 years. The 50 healthy controls were enrolled in the physical health center of Zhongshan Hospital, Fudan University and their physical parameters were within normal range. The 50 healthy controls included 27 males and 23 females, and the age range was 37–64 years and the mean age was 46.3 ± 5.1 years. These healthy controls were selected from the suspicious patients who received aortic biopsy for the diagnosis of suspicious clinical disorders, such as takayasu arteritis, while those suspicious clinical disorders were finally excluded. The aim of the selection of these patients was to match the age and gender distribution of patient group. All participants signed informed consent. The present study was approved by Ethics Committee of Zhongshan Hospital, Fudan University before the admission of patients.

### Specimens and cells

To perform *in vivo* analysis, aortic biopsy was performed and aortic media specimen (collected after the resected biopsies were dissected) was obtained from each participant.

To perform *in vitro* analysis, human aortic smooth muscle cells (HAoSMC, PromoCell) were cultivated with medium 231 in an incubator (37°C, 5% CO_2_).

### RNA extraction and qRT-PCR

Aortic media specimens were ground in liquid nitrogen and RNAzol reagent was added to extract total RNAs. HAoSMCs were also directly mixed with RNAzol reagent to extract total RNAs. Reverse transcription was performed using SuperScript III Reverse Transcriptase (Thermo Fisher Scientific) to synthesize cDNA. After that, PCR reaction systems were prepared using Applied Biosystems™ Power™ SYBR™ Green Master Mix with 18S rRNA as endogenous control to detect the expression of LOXL1-AS and Giver. All data normalizations were performed based on 2^−∆∆*C*_T_^ method.

### Transient transfection

LOXL1-AS and Giver full length genomic DNAs were inserted into pcRNA3.1 vector (Sangon, Shanghai, China) to establish LOXL1-AS and Giver expression vectors. Giver siRNA and negative control siRNA were designed by Sangon (Shanghai, China). Lipofectamine 2000 (Invitrogen, Carlsbad, U.S.A.) reagent was used to perform cell transfections with 10 nM vectors and 35 nM siRNAs. Cells were collected 24 h after transfection to perform subsequent experiments.

### Cell proliferation assay

Cells were harvested at 24 h after transection and singles cell suspensions (3 × 10^4^ cells/1 ml) were prepared. Cells were cultivated in a 96-well plate with 0.1 ml cell suspension in each well. Cells were cultivated under normal conditions (37°C, 5% CO_2_), and CCK-8 solution (10 μl, Sigma–Aldrich) was added every 4 h before the end of cell culture. After the addition of 10 μl DMSO, OD values (450 nm) were measured.

### Cell apoptosis assay

After transfection, 4 × 10^6^ cells were treated with trypsin. After washing with precooled PBS buffer without calcium and magnesium, cells were mixed with 100 μl binding buffer which followed by incubation for 10 min in the dark. After that, 6 μl of Annexin V-FITC and 10 μl of PI stain (MA0220, Meilun Bio, China) was added and cells were incubated in dark for 20 min. Finally, apoptotic cells were detected by flow cytometry.

### Western blot

HAoSMCs were harvested and RIPA (Sangon, Shanghai, China) was used to extract proteins. Proteins were denatured and 10% SDS/PAGE gel was used to perform electrophoresis. After gel transfer (PVDF membrane) and blocking (FBS containing 5% non-fat milk) for 2 h, blotting was performed using rabbit primary antibodies of Bcl-2 (1:1200, ab59348, Abcam) and GAPDH (1:1200, ab9485, Abcam), as well as secondary antibody of HRP goat anti-rabbit (IgG) (1:1000; ab6721; Abcam). ECL detection reagent (EMD Millipore) was used for signal development, and ImageJ v1.46 software was used to normalize gray values.

### Statistical analysis

Each experiment included three biological repeats. GraphPad Prism 6 software was used to process all data. Unpaired t test was used for the comparisons between patient and control groups. ANOVA (one-way) and Tukey test were used for comparisons amongst different cell treatment groups. ROC curve analysis was performed with TAA patients as true positive cases and healthy controls as true negative cases. Linear regression was performed to analyze the correlation between Giver and LOXL1-AS. *P<*0.05 was the cutoff value of statistically significant.

## Results

### LOXL1-AS was up-regulated in TAA patients

**Figure 1:**
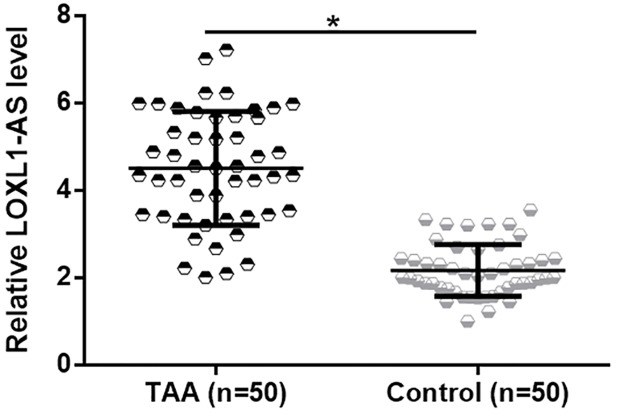
LOXL1-AS was up-regulated in TAA patients RT-qPCR results showed that expression levels of LOXL1-AS in aortic media specimens were significantly higher in TAA patients than in healthy control group (*P<0.05).

RT-qPCR was performed to evaluate the differential expression of LOXL1-AS in TAA patients and healthy control group. It was observed that the expression levels of LOXL1-AS in aortic media specimens were significantly higher in TAA patients than in healthy control group (Figure 1, *P<*0.05), suggestive of the involvement of LOXL1-AS in TAA.

### Altered expression levels of LOXL1-AS distinguished TAA patients from healthy controls

**Figure 2:**
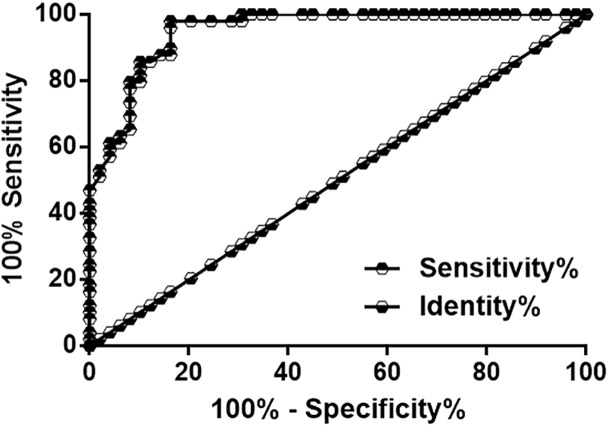
Altered expression levels of LOXL1-AS distinguished TAA patients from healthy controls ROC curve analysis showed that altered expression levels of LOXL1-AS distinguished TAA patients from healthy controls.

ROC curve analysis was performed with TAA patients (*n*=50) as true positive cases and healthy controls (*n*=50) as true negative cases to evaluate the diagnostic value of LOXL1-AS expression for TAA. The results showed that the area under the curve was 0.95 (standard error: 0.020, 95% CI: 0.91–0.99, Figure 2).

### LncRNA Giver was up-regulated in TAA patients and positively correlated with LOXL1-AS

**Figure 3:**
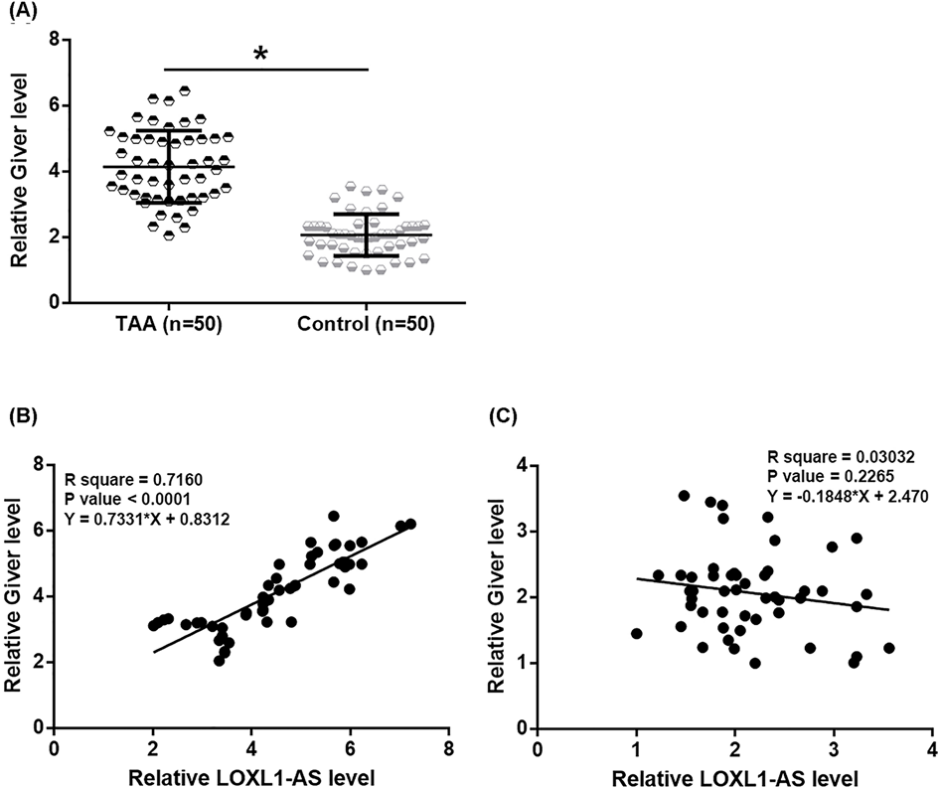
LncRNA Giver was up-regulated in TAA patients and positively correlated with LOXL1-AS RT-qPCR showed that Giver was also up-regulated in TAA patients than in healthy controls in aortic media specimens (**A**), and was positively correlated with LOXL1-AS in TAA patients (**B**), but not in healthy control (**C**), (*P<0.05).

RT-qPCR was also performed to evaluate the differential expression of Giver in TAA patients and healthy control group. It was observed that the expression levels of Giver in aortic media specimens were also significantly higher in TAA patients than in healthy control group (Figure 3A, *P*<0.05). Linear regression was performed to analyze the correlation between Giver and LOXL1-AS. It was observed that expression levels of these two lncRNAs were positively and significantly correlated in TAA patients (Figure 3B). However the correlation between the two lncRNAs was not significant in healthy controls (Figure 3C).

### LOXL1-AS overexpression mediated the up-regulation of Giver in HAOSMC

**Figure 4:**
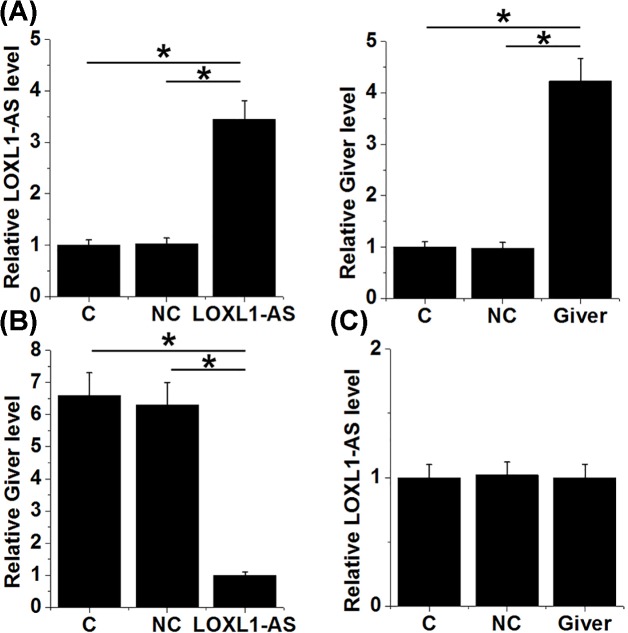
LOXL1-AS overexpression mediated the up-regulation of Giver in HAoSMC LOXL1-AS and Giver expression were overexpressed at 24 h after transfection (**A**). In addition, LOXL1-AS overexpression mediated the up-regulation of Giver in HAoSMC (**B**), while Giver overexpression failed to significantly affect LOXL1-AS (**C**), (*P<0.05).

In order to further investigate the interaction between LOXL1-AS and Giver, LOXL1-AS and Giver expression vectors were transfected into HAOSMCs. After transfection, LOXL1-AS and Giver expression were significantly up-regulated comparing to control (C) and negative control (NC, empty vector transfection) groups (Figure 4A, *P*<0.05). In addition, LOXL1-AS overexpression mediated the up-regulation of Giver in HAOSMC (Figure 4B, *P*<0.05), while Giver overexpression failed to significantly affect LOXL1-AS (Figure 4C).

### LOXL1-AS regulated the proliferation and apoptosis of HAOSMC through Giver

**Figure 5:**
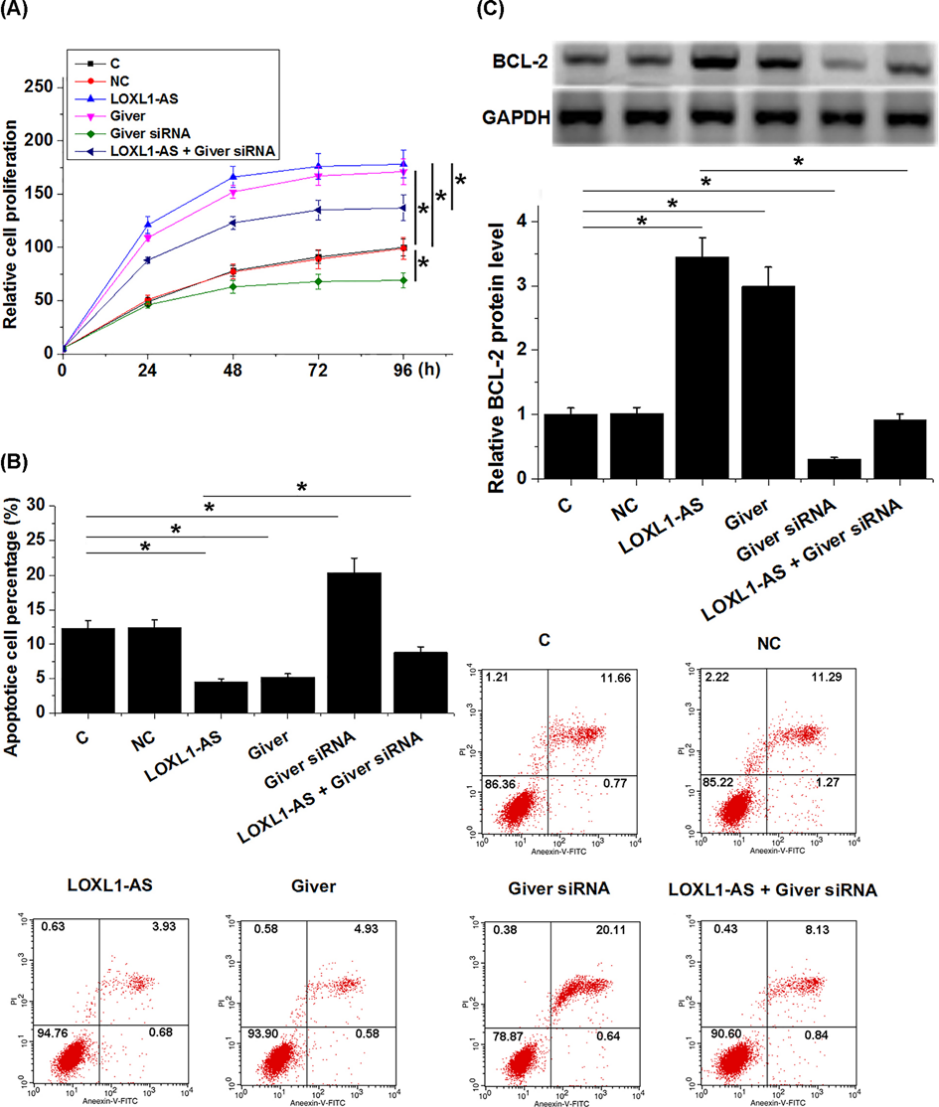
LOXL1-AS regulated the proliferation and apoptosis of HAOSMC through Giver LOXL1-AS and Giver overexpression resulted in promoted proliferation (**A**) and inhibited apoptosis (**B**) of HAOSMC. Giver silencing played an opposite role and attenuated the effect of LOXL1-AS overexpression. Moreover, LOXL1-AS and Giver overexpression resulted in up-regulated antiapoptosis Bcl-2 expression, and. Giver silencing played an opposite role and attenuated the effect of LOXL1-AS overexpression (**C**), (*P<0.05).

Comparing with control (C) and NC groups, LOXL1-AS and Giver overexpression resulted in promoted proliferation (Figure 5A, *P<*0.05) and inhibited apoptosis (Figure 5B, *P<*0.05) of HAOSMC. Giver silencing played an opposite role and attenuated the effect of LOXL1-AS overexpression. Moreover, LOXL1-AS and Giver overexpression resulted in up-regulated antiapoptosis Bcl-2 expression, and Giver silencing played an opposite role and attenuated the effect of LOXL1-AS overexpression (Figure 5C).

## Discussion

LOXL1-AS has been demonstrated as an oncogenic lncRNA in medulloblastoma [13]. Our study investigated the role of LOXL1-AS in TAA. Our study is the first to report that LOXL1-AS was up-regulated in TAA patients, and LOXL1-AS may promote TAA by up-regulating Giver.

The involvement of lncRNAs in TAA has been reported by several previous studies. Wang et al. reported that lncRNA HIF1A-AS1 interacted with BRG1 to regulate the apoptosis and proliferation of vascular smooth muscle cells, thereby participating in the development of TAA [9]. In another study, lncRNA MALAT1 was also reported to interact with BRG1 to regulate the function of smooth muscle in TAA [14]. Moreover, a recent microarray study revealed a large number of differentially expressed lncRNAs in TAA [15]. Giver participates in inflammation, oxidative stress, and proliferation in vascular smooth muscle cells, which are involved in TAA [12]. Therefore, it will be reasonable to hypothesize that Giver may also be involved in TAA. Our study showed that Giver was up-regulated in TAA, and positively regulated the proliferation and negatively regulate the apoptosis of HAOSMC, suggestive of the enhancing effect of Giver on the progression of TAA.

Our preliminary deep sequencing suggested the positive correlation between Giver and LOXL1-AS in TAA. It is known that lncRNAs participate in human diseases mainly by interacting with downstream effector pathways [16,17]. Studies on the interaction between different lncRNAs are rare. Our data suggest that LOXL1-AS is an upstream activator of Giver in HAOSMC, and this interaction between two lncRNAs is involved in the regulation of the proliferation and apoptosis of HAOSMC. Our study may provide new insights to the pathogenesis of the TAA. However, the molecular mechanism underlying this interaction is unclear. It is likely that the interaction between LOXL1-AS and Giver is mediated by certain pathways activated in TAA due to the fact that LOXL1-AS and Giver are only significantly correlated in TAA patients but not in healthy controls.

In conclusion, LOXL1-AS was overexpressed in TAA and may up-regulate Giver to promote TAA.

## Availability of Data and Materials

The analyzed datasets generated during the study are available from the corresponding author on reasonable request.

## Abbreviations

HAoSMC: human aortic smooth muscle cell
lncRNA: long non-coding RNA
NC: negative control
TAA: thoracic aortic aneurysm
